# Freestanding Films of Reduced Graphene Oxide Fully Decorated with Prussian Blue Nanoparticles for Hydrogen Peroxide Sensing

**DOI:** 10.1021/acsomega.4c01457

**Published:** 2024-07-10

**Authors:** Vitor H. N. Martins, Monize M. da Silva, Daniel A. Gonçalves, Volker Presser, Samantha Husmann, Victor H. R. Souza

**Affiliations:** † Faculty of Exact Science and Technology, Universidade Federal da Grande Dourados, Dourados, Mato Grosso do Sul 79804-970, Brazil; ‡ INM—Leibniz Institute for New Materials, Campus D2-2, 66123 Saarbrücken, Germany; § Department of Materials Science & Engineering, Saarland University, Campus D2 2, 66123 Saarbrücken, Germany; ∥ Saarene—Saarland Center for Energy Materials and Sustainability, Campus C4 2, 66123 Saarbrücken, Germany

## Abstract

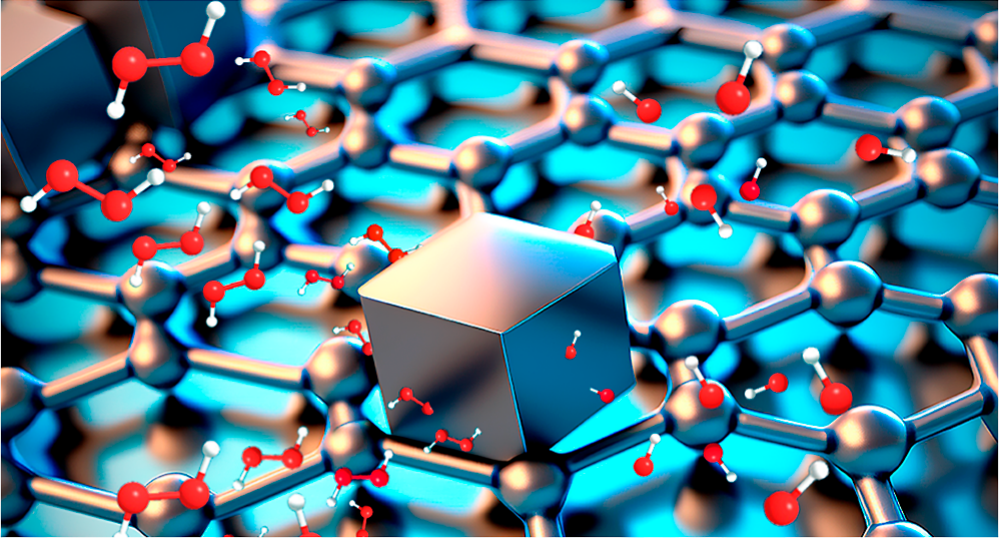

Developing thin, freestanding electrodes that work simultaneously as a current collector and electroactive material is pivotal to integrating portable and wearable chemical sensors. Herein, we have synthesized graphene/Prussian blue (PB) electrodes for hydrogen peroxide detection (H_2_O_2_) using a two-step method. First, an reduced graphene oxide/PAni/Fe_2_O_3_ freestanding film is prepared using a doctor blade technique, followed by the electrochemical deposition of PB nanoparticles over the films. The iron oxide nanoparticles work as the iron source for the heterogeneous electrochemical deposition of the nanoparticles in a ferricyanide solution. The size of the PB cubes electrodeposited over the graphene-based electrodes was controlled by the number of voltammetric cycles. For H_2_O_2_ sensing, the PB10 electrode achieved the lowest detection and quantification limits, 2.00 and 7.00 μM, respectively. The findings herein evidence the balance between the structure of the graphene/PB-based electrodes with the electrochemical performance for H_2_O_2_ detection and pave the path for developing new freestanding electrodes for chemical sensors.

## 1 Introduction

The market for portable and wearable chemical sensors has rapidly increased over the last few years as a tool to bring more convenience to human life and to meet the need for quick responses without using sophisticated equipment.^1,2^ As a consequence, developing new materials capable of integrating such gadgets and providing suitable monitoring performance is pivotal to new advancements in this field.

Many features are required from the materials used in wearable sensors, but flexibility and (electrical or thermal) conductivity are often essential ones. Graphene-based composites have been extensively explored for this purpose owing to the outstanding properties of graphene (e.g., tunable conductivity, high surface area, and controllable thickness)^3^ to prepare composites with different classes of compounds aiming for further application in sensor devices, such as nanoparticles or molecularly imprinted polymers.^4−7^ In addition, graphene-based composites may be processed as paper-like electrodes,^8,9^ thin and freestanding materials that can work as a current collector and chemical detector. Such paper-like graphene-based electrodes are easily fabricated through different techniques, ranging from vacuum filtration [in which graphene-based films are assembled onto a membrane surface during the vacuum drying process of graphene oxide (GO) or reduced graphene oxide (rGO) dispersion] to evaporation, electrochemical methods, electrospraying, or roll-to-roll processing.^10^ Using a doctor blade technique, we recently synthesized freestanding films of rGO with polyaniline (PAni).^11^ A high-viscous mixture of GO and PAni is deposited over a glass substrate and further exposed to hydrazine vapor, resulting in free-standing films with tunable conductivity.

The performance of graphene-based films as chemical sensors may be improved by preparing composites with electrocatalytic and/or selective materials. The electrochemical detection of low levels of hydrogen peroxide (H_2_O_2_), a molecule related to several pathological and physiological processes and associated with a gamut of diseases,^12^ has been recognizably achieved using graphene/Prussian blue (PB) composites.^13,14^ PB is a hexacyanoferrate with a face-centered cubic structure, which has ferrous (Fe^2+^) and ferric (Fe^3+^) metallic species in alternation, which coordinate to carbon and nitrogen atoms, respectively, from cyanide ligand groups.^15^ PB has different redox states and is found in three oxidized forms: Berlin green and Prussian yellow, and the most reduced, Prussian white, which has enhanced electrocatalytic performance toward H_2_O_2_ reduction.^16^ Such activity occurs through the diffusion of H_2_O_2_ molecules into the small vacancies formed by PB porous lattices, enabling the interaction with ferrous species. The similarity in size between H_2_O_2_ and PB vacancies also leads to significant electrode selectivity. Additionally, PB is a biocompatible material.^17^ These combined characteristics led to the extensive application of PB in biosensing, metal-ion batteries, and wearables fields.^18,19^

Among several methods to synthesize graphene/PB-based composites, the electrochemical deposition of PB by a heterogeneous reaction of ferrocyanide ions in aqueous media and iron-based species encapsulated into the carbon nanostructures has emerged as a strategy to synthesize materials with tunable properties.^20−22^ Moreover, the effective interaction between PB nanoparticles and carbon-based materials improves the electrochemical stability of PB and provides low limits of detection (LDs) for H_2_O_2_.

Herein, we have synthesized freestanding rGO/PB electrodes for H_2_O_2_ electrochemical detection through a two-step method. First, the freestanding electrode was prepared by exposing a GO/PAni/Fe^3+^ film to a hydrazine vapor, resulting in an rGO/PAni/Fe_2_O_3_ freestanding and electrically conductive film. Afterward, we used the films as both working electrodes and iron sources for the electrochemical deposition of PB over the film surface. We have designed freestanding electrodes with different structural compositions and morphologies by controlling the cyclic voltammetry (CV) cycles. The films were further investigated for H_2_O_2_ detection. The performance of the electrodes as electrochemical sensors was strictly related to the size of PB cubes on the graphene surface, evidencing the importance of fine-tuning film morphology for sensor applications.

## 2 Experimental Section

### 2.1 Synthesis of GO and Polyaniline

GO was synthesized using a modified Hummers method, as described elsewhere.^23^ Graphite flakes (Grafine, Nacional de Grafite) were preoxidized in a mixture of potassium persulfate (K_2_S_2_O_8_, 99%, Sigma-Aldrich) and phosphorus pentoxide (P_2_O_5_, 99%, Sigma-Aldrich) dissolved in concentrated sulfuric acid (H_2_SO_4_, 96%, Sigma-Aldrich) at 80 °C. The mixture was cooled, diluted in deionized water, and filtered. The solid was washed with deionized water and air-dried, and then the preoxidized graphite was mixed with concentrated sulfuric acid under constant magnetic stirring at 0 °C in an ice bath. Potassium permanganate salt (KMnO_4_, 99%, Sigma-Aldrich) was added, followed by hydrogen peroxide (30% by volume, Sigma-Aldrich). The dispersion was washed with 3.4% hydrochloric acid, and the product was filtered and dried, followed by washing with acetone, and, finally, filtered and dried in air.

PAni was synthesized using a rapid mixing method, as described elsewhere.^24^ For this purpose, 16 mmol of aniline (99%, Sigma-Aldrich) was dissolved in 50 mL of 1 mol L^–1^ HCl solution and mixed with 4 mmol of ammonium peroxydisulfate [(NH_4_)_2_S_2_O_8_, 98%, Sigma-Aldrich] previously dissolved in 50 mL of 1 mol L^–1^ HCl. The solution was stirred immediately after mixing. PAni (green emeraldine salt) was washed with ammonium hydroxide solution and deionized water until pH 7, where emeraldine base PAni was obtained.

### 2.2 Synthesis of the Freestanding Films

The freestanding electrodes were produced following a similar method reported earlier.^11^ A high-viscosity gel was prepared by mixing 2.5 mL of GO dispersion (40 mg mL^–1^) with 2.5 mL of polyaniline emeraldine base/iron chloride dispersion (5 mg mL^–1^ of each compound). This was mechanically stirred using a vortex mixer until a viscous, homogeneous green mixture formed. The final blend was deposited as a uniform film onto glass substrates using a doctor blade. Finally, the film was dried at 60 °C for 30 min, followed by chemical steam reduction using hydrazine vapor, resulting in the freestanding film (Supporting Information, Figure S1). This sample was named rGO/PAni/Fe_2_O_3_.

### 2.3 Electrochemical Deposition of PB Nanoparticles

**Scheme 1:**
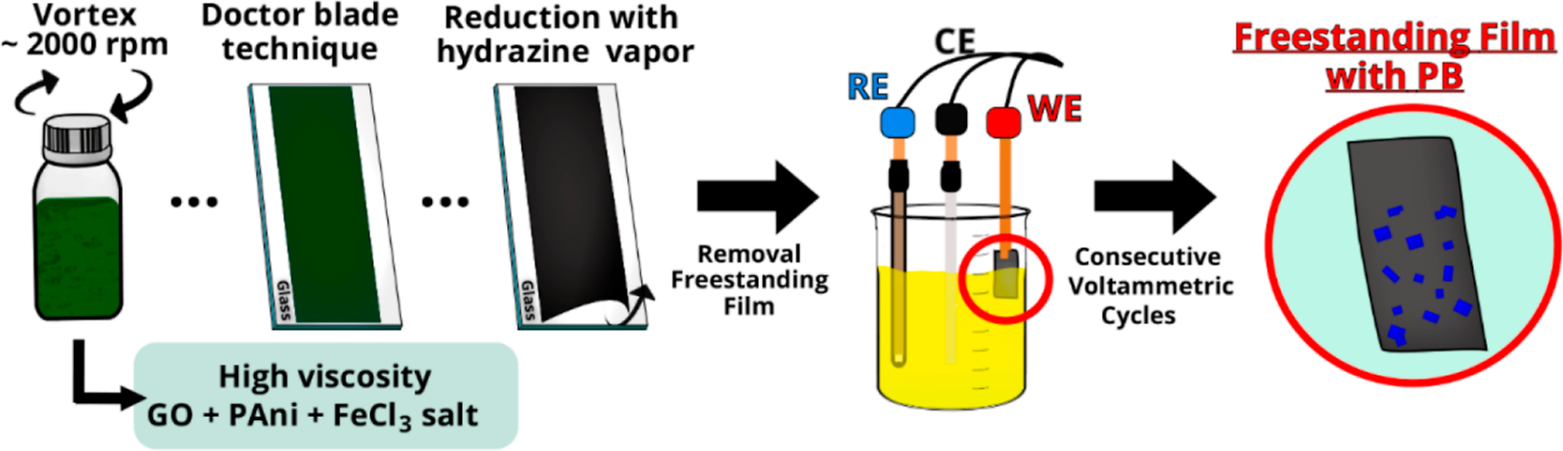
Schematic Representation of PB Electrodeposition over the Free-Standing Graphene-Based Electrodes

The electrochemical modification of the rGO/PAni/Fe_2_O_3_ freestanding films was performed by CV using a conventional three-electrode electrochemical cell. The schematic representation of the process is depicted in Scheme 1. An Ag|AgCl (KCl 3 mol L^–1^) electrode, a platinum plate, and the freestanding rGO/PAni/Fe_2_O_3_ films were used as a reference, counter, and working electrode, respectively. In short, 0.5 cm^2^ of the working electrode was immersed into a 1 mmol L^–1^ K_3_[Fe(CN)_6_]/0.1 mol L^–1^ KCl solution. A sweep potential from −0.3 to 1.4 V (vs Ag|AgCl, KCl 3 mol L^–1^) at 50 mV s^–1^ rate was adopted, varying the number of voltammetric cycles (5, 10, 25, 100, and 200 cycles). After the electrochemical modification, the electrodes were rinsed with deionized water and dried at 60 °C for 30 min. Samples were named PB5, PB10, PB25, PB100, and PB200.

### 2.4 Materials Characterization

Scanning electron microscopy (SEM) was carried out with a Gemini 500 (ZEISS) at a voltage of 2 kV and an in-lens secondary electron detector, coupled with an energy-dispersive X-ray (EDX) spectroscopy detector (Oxford Instruments) for elemental analysis and a SEM-FEG/Tescan instrument with a voltage of 10 kV. An X-ray diffractometer D8 Discover by Bruker AXS with Cu Kα (λ = 1.5406 Å, 40 kV, 40 mA) and a Göbel mirror, 1 mm from the focal point, and a VANTEC-500 detector were utilized to determine the crystalline structure of the electrodes. A Renishaw InVia Raman microscope using a HeNe excitation laser (633 nm) with 0.5 mW laser power on the sample was used to acquire the Raman spectra. Fourier transform infrared (FTIR-Jasco 4100) spectra using the attenuated total reflectance mode were used in a range of 700–2000 cm^–1^. The electrochemical measurements were performed in an Autolab potentiostat versus an Ag|AgCl reference electrode (3 mol L^–1^ KCl), a platinum plate as the counter electrode, and the freestanding films as the working electrode (1.5 cm × 0.5 cm). Around 0.5 cm^2^ of the working electrodes was immersed in the electrolyte.

All electrochemical measurements were performed in a glass electrochemical cell (50 mL) at room temperature using a portable bipotentiostat/galvanostat μ-Autolab Type III (Metrohm Autolab) with the aid of NOVA 2.15 software. A standard solution using 0.01 mol L^–1^ stock solutions of H_2_O_2_ in ultrapure water (resistance ≥ 18.2 MΩ cm, OS 20 LTXE Gehaka) was used to prepare the calibration curve and spiked samples during addition–recovery experiments. All the H_2_O_2_ solutions contained 0.1 mol L^–1^ KCl and a 0.1 mol L^–1^ PBS buffer (K_2_HPO_4_/KH_2_PO_4_, pH 7.0, Neon). A micropipette of 10–100 μL was used for analytical performance. The effect of interference on the detection was carried out with the addition of glucose (Glu), uric acid (UA), and ascorbic acid (AA) from 0.01 mol L^–1^ stock solutions and prepared synthetic urine samples.^25^ The amperometric detection was carried out in a three-electrode system by applying −0.1 V (vs Ag|AgCl, KCl 3 mol L^–1^) at an rGO/PB freestanding film as the work electrode in contact with stirred solutions (400 rpm) of different hydrogen peroxide (H_2_O_2_) concentrations. A Pt planar was used as an auxiliary electrode. For multiple H_2_O_2_ detections (every 24 h), the PB10 electrode was washed and dried at room temperature after each measurement. The CV curves at different scan rates (5, 10, 15, 20, 30, 40, and 50) were obtained using K_3_[Fe(CN)_6_]/K_4_[Fe(CN)_6_] solution (10 mM concentration, 1:1 molar ratio) containing 0.5 mol L^–1^ of KCl.

## 3 Results and Discussion

### 3.1 Electrochemical Design of the Graphene-Based Electrodes

Designing freestanding graphene-based electrodes that work as both current collectors and electroactive materials for detecting different analytes requires fine-tuning of the chemical and electrochemical properties of the composites. In previous work, we have shown that freestanding rGO/PAni/Fe_2_O_3_ can be synthesized by exposing GO/PAni/Fe^3+^ films to hydrazine vapor.^11^ The presence of the conducting polymer in the emeraldine base form as a binder is essential to film formation. In addition, the double function of hydrazine in reducing GO to rGO and producing Fe_2_O_3_ nanoparticles in the rhombohedral phase from Fe^3+^ ions was also observed.

**Scheme 2:**
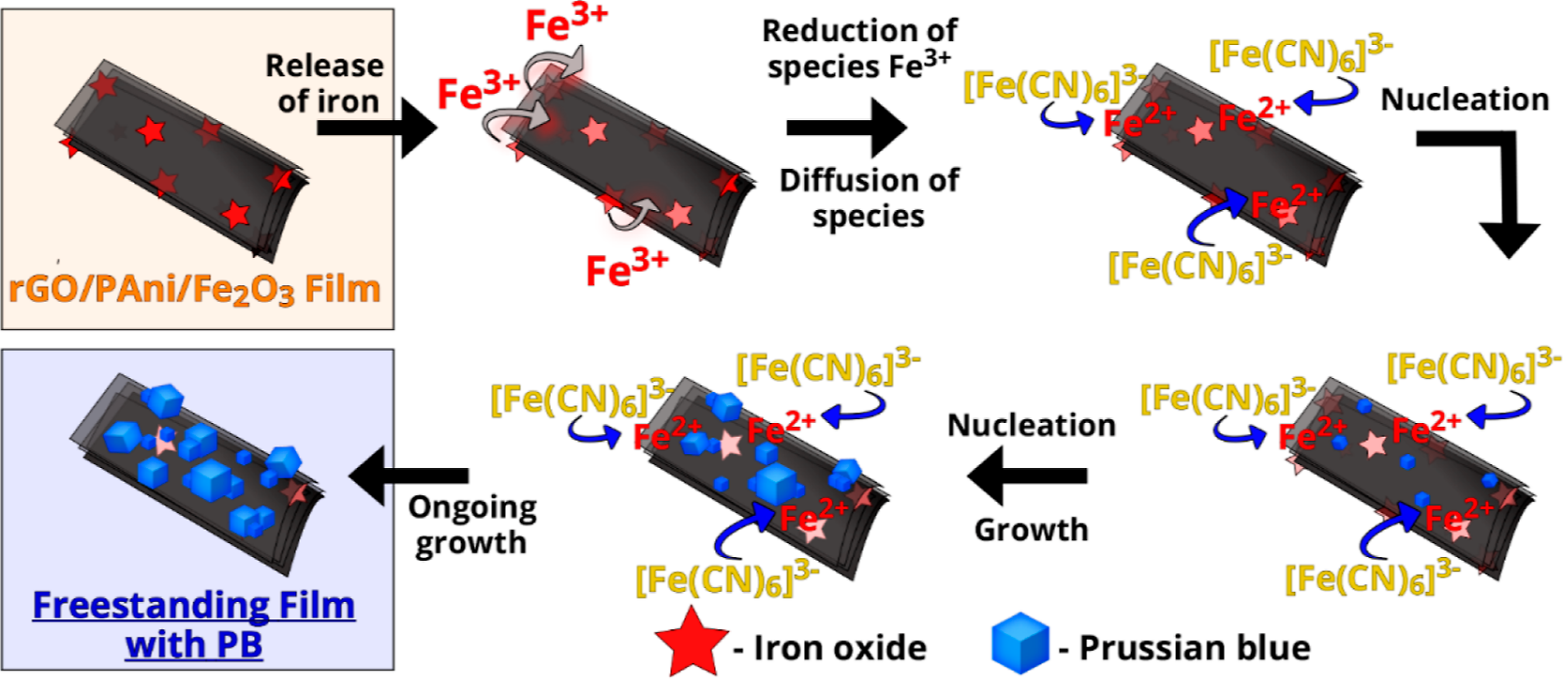
Schematic Representation of the Mechanism for PB Electrodeposition over the Freestanding Films

Among the methods to synthesize PB, electrosynthesis through the heterogeneous reaction of iron-based species encapsulated into carbon-based materials (carbon nanotubes and graphene) controls the size and amount of PB and PB-analogue nanoparticles growing over the carbon surface.^15,20,22,26^ Throughout this approach, transition metallic ions are released from inside the carbon nanostructure film over the voltammetric cycles, allowing the electrodeposition of PB nanoparticles all over the graphene-based electrode surface. The mechanism for the electrodeposition of PB nanoparticles over the freestanding films is depicted in Scheme 2. Moreover, this method allows a fine-tuning of the PB/carbon nanostructure interaction, which is crucial for further electrochemical properties and stability of the graphene/PB composites.

**Figure 1:**
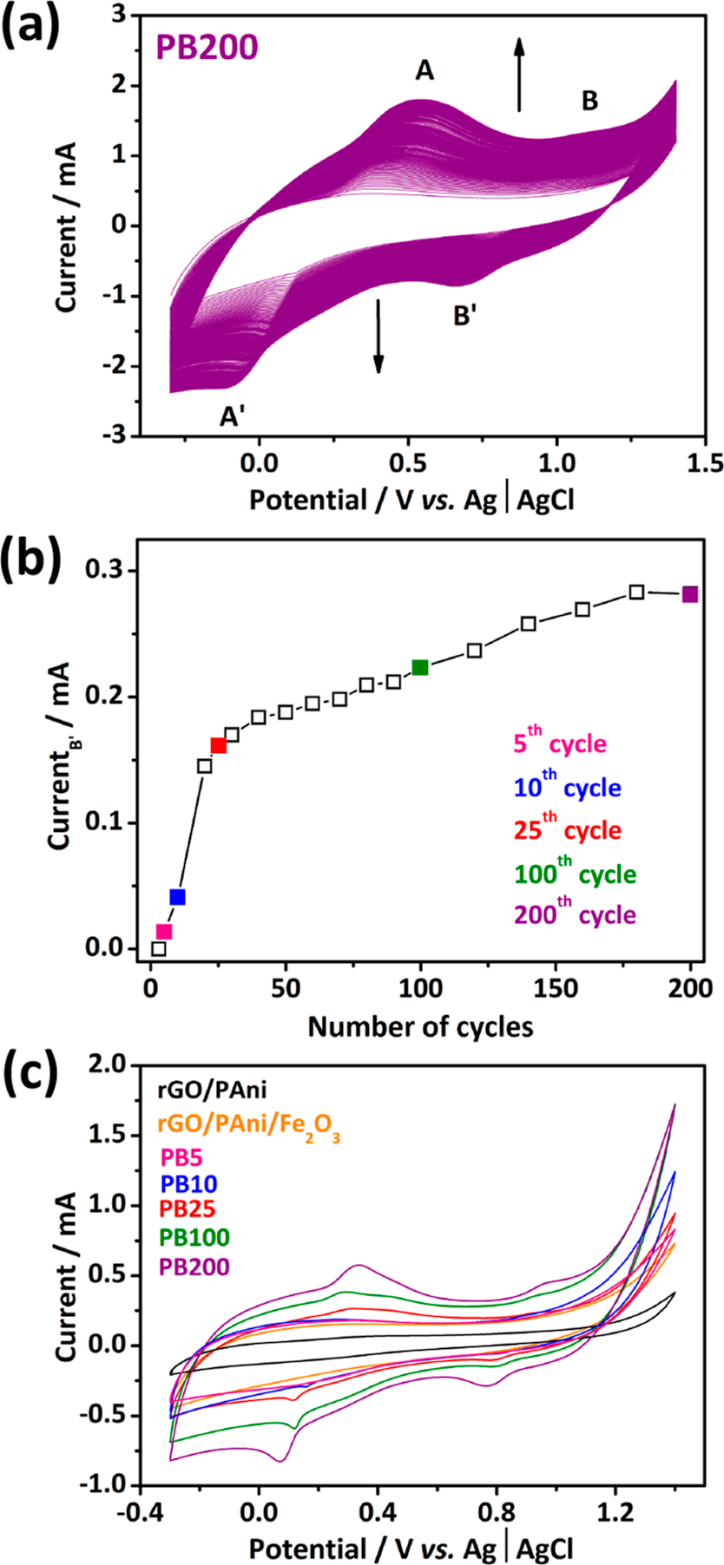
PB electrodeposition throughout 200 CV cycles in an aqueous 1 mmol L^–1^ K_3_[Fe (CN)_6_]/0.1 mol L^–1^ KCl solution at 50 mV s^–1^ (a). The intensity of the cathodic peak at 0.7 V as a function of CV cycles (b). CV curves at 10 mV s^–1^ of the different materials in a neutral electrolyte, KCl 0.1 mol L^–1^ (c).

The main advantage of the rGO/PAni/Fe_2_O_3_ freestanding film is its dual function as a current collector and iron source for the heterogeneous electrodeposition of PB. Figure 1a displays the electrodeposition profile throughout 200 CV cycles at 50 mV s^–1^ of the rGO/PAni/Fe_2_O_3_ electrode in 1 mmol L^–1^ K_3_[Fe (CN)_6_]/0.1 mol L^–1^ KCl solution. Two redox pairs around 0.55/–0.10 V and 1.10/0.68 V (vs Ag|AgCl, KCl 3 mol L^–1^) are noticed, corresponding to conversion throughout the different oxidized/reduced forms of PB.^20^ The redox peak intensity gradually increases with the CV cycles (Figure 1b). A steep increase in the current intensity occurs in the first 25 cycles, followed by steady increments until 200 cycles, corresponding to a continuous PB electrodeposition onto the graphene-based electrode. The pattern in Figure 1b indicates that after 200 cycles, the electrode’s surface is close to saturation. To design composites aiming for better electrochemical detection of H_2_O_2_, we have synthesized PB/graphene-based composites by varying the number of CV cycles, which will be named PB5, PB10, PB25, PB100 (Supporting Information, Figure S2a–c), and PB200.

The cyclic voltammograms in aqueous KCl 0.1 mol L^–1^ of the different electrodes are shown in Figure 1c. A non-Faradaic current is observed for rGO/PAni and rGO/PAni/Fe_2_O_3_ electrodes, wherein no redox peak is clearly observed. A higher capacitive response is noticed for rGO/PAni/Fe_2_O_3_, which may be associated with the pseudocapacitive characteristic of the Fe_2_O_3_ species. A different behavior is observed for composites with PB, with redox processes characteristic of PB. A first redox pair was observed around 0.34/0.06 V (vs Ag|AgCl, KCl 3 mol L^–1^), associated with Fe^II^[Fe^II^(CN)_6_]^2–^/Fe^III^[Fe^II^(CN)_6_]^−^ processes in PB. A second pair around 0.98/0.76 V (vs Ag|AgCl, KCl 3 mol L^–1^) is associated with the redox process of Fe^III^[Fe^II^(CN)_6_]^−^/Fe^III^[Fe^III^(CN)_6_]. In addition, a progressive increase in the intensity of these pairs when comparing the electrodes with 5, 10, 25, 100, and 200 cycles is noticed, giving evidence that as the number of cycles increased, the more significant the electrochemical contribution of PB was.

### 3.2 Morphological and Structural Characterization

**Figure 2:**
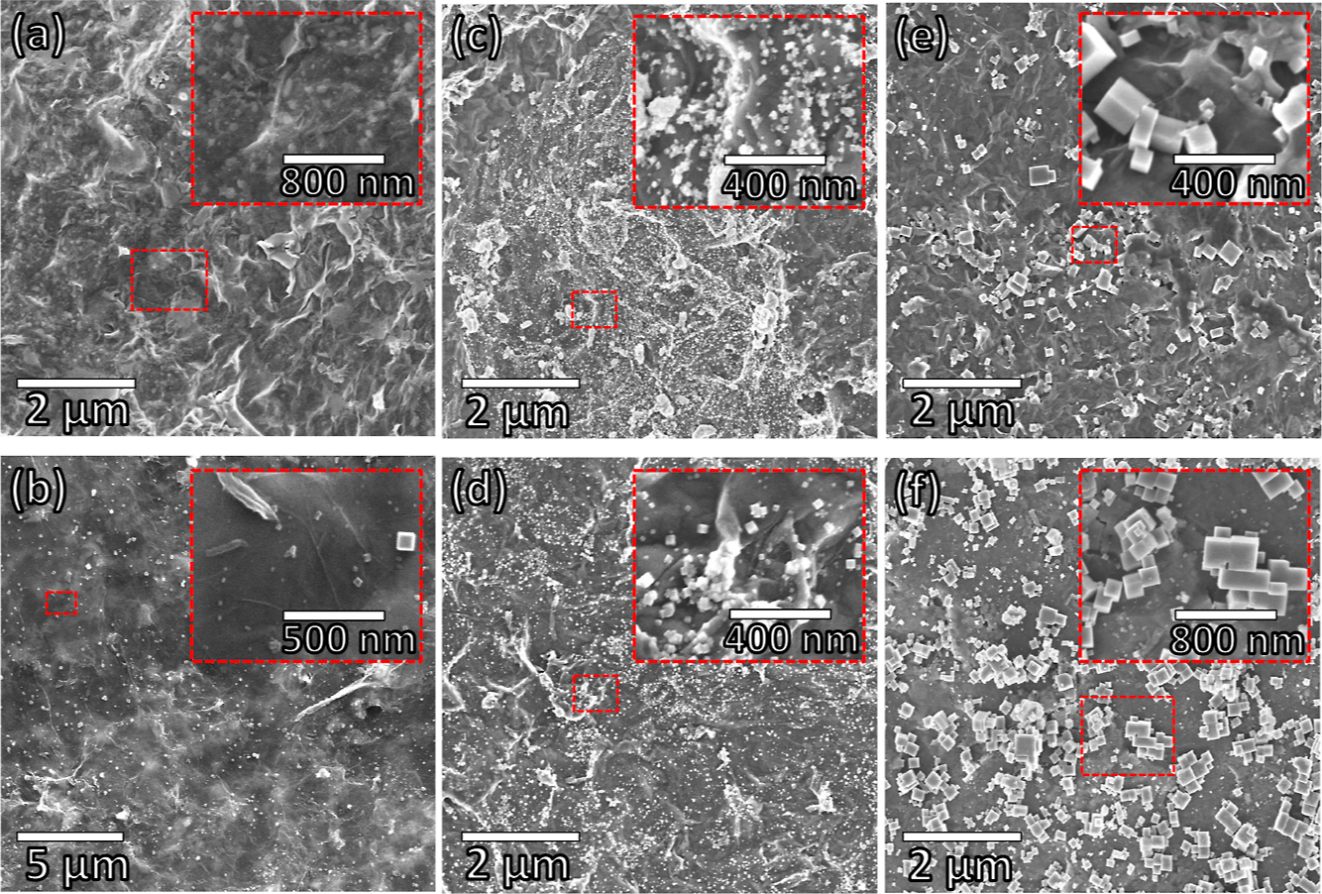
Scanning electron micrographs of (a) rGO/PAni/Fe_2_O_3_, (b) PB5, (c) PB10, (d) PB25 (e) PB100, and (f) PB200, along with the high magnification images in insets.

An evolution of the freestanding electrode morphology as a function of the PB electrodeposition is depicted in Figure 2. The composite electrode surfaces differ from bare electrodes (rGO/PAni/Fe_2_O_3_ in Figure 2a and rGO/PAni in Figure S3 in Supporting Information). The rGO/PAni electrode exhibits the wrinkled graphene sheet morphology throughout the material’s surface, with polyaniline fibers at some points (Figure S3; green arrows). Similarly, the rGO/PAni/Fe_2_O_3_ electrode shows the presence of rGO and PAni. Moreover, this material also exhibits a homogeneous distribution of brighter, small particles corresponding to Fe_2_O_3_ nanoparticles (Figure 2a inset).^11^ In PB5, nanometric PB cubes are dispersed on the graphene surface (Figure 2b). An increase in the PB cubes is evident toward more CV cycles (from PB10 in Figure 2c to PB25 in Figure 2d), and cube agglomeration is also noticed, especially in the PB200 sample. This is well observed in the size distribution of the PB cubes (Supporting Information, Figure S4). PB cubes’ sizes range from 27 ± 12 to 47 ± 20, 78 ± 30, 154 ± 86, and 335 ± 265 nm in samples PB5, PB10, PB25, PB100, and PB200, respectively. Figure S5 shows the cross-sectional images of the freestanding film before (a) and after (b) the electrodeposition of PB particles throughout a hundred cycles (PB100). The presence of PB cubes exclusively on the surface of the electrode is noticed, which may be relevant for the electrochemical detection of H_2_O_2_.

**Figure 3:**
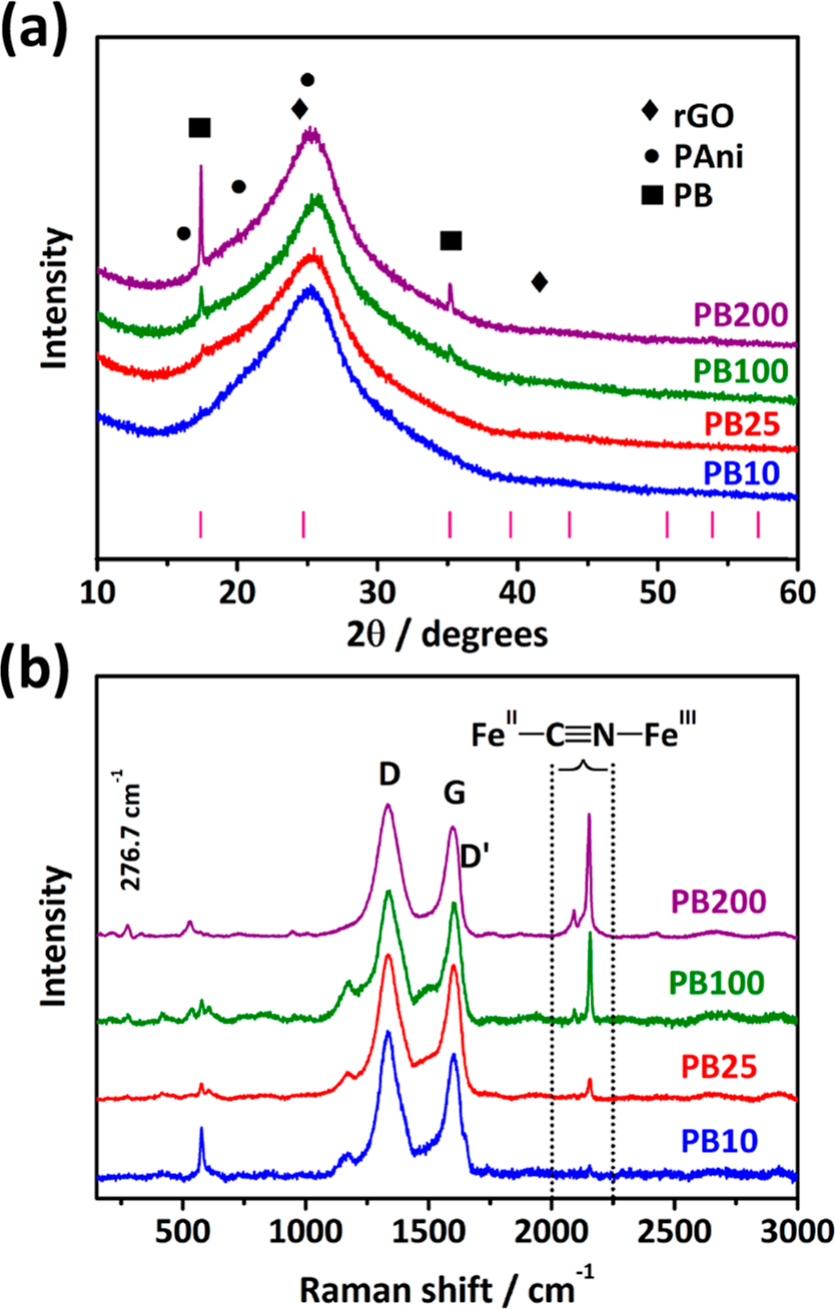
X-ray diffractograms (a) and Raman spectra (λ = 633 nm) (b) collected from samples PB10 (blue line), PB25 (red line), PB100 (green line), and PB200 (purple line).

PB formation was also confirmed by X-ray diffraction and Raman spectroscopy, as shown in Figure 3. The X-ray diffractograms in Figure 3a exhibit an intense peak at 23.1° 2θ for all composites, characteristic of the crystallographic planes (002) of graphene, indicating an amorphous organization resulting from a random stacking of the rGO sheets.^27^ The films decorated with PB present new well-defined peaks at 17.4° 2θ and 35.2° 2θ corresponding to the (200) and (400) planes of cubic PB, respectively.^21,28^ In addition, an increase in the PB peak intensity is noticed along with CV cycles, indicating a higher contribution of PB, as observed from the scanning electron micrographs.

The Raman spectra show the presence of characteristic bands of rGO, PAni, and PB. Bands D and D′ at 1336 and 1613 cm^–1^, respectively, refer to structural defects in rGO (occurrences of 5-membered and 7-membered rings, border defects).^29−31^ The G band at 1596 cm^–1^ is characteristic of the sp^2^-hybridized carbon material. In addition, bands at 1171 and 1225 cm^–1^ refer to the C≡N stretch in diimine units in the benzenoid ring, and 1470 and 1513 cm^–1^ correspond to the C=N stretch in quinoid ring units, all four characteristics of the emerald salt form of PAni, thus indicating an acidic protonation produced by GO in the polymer.^32^ The presence of PAni is also confirmed through FTIR analysis (Figure S6), with all peaks corresponding to the protonated form of the conducting polymer.^11^ As observed by X-ray diffraction in Figure 3a, the bands corresponding to PB also increase with CV cycles. The band at 276 cm^–1^ corresponds to Fe–C≡N deformation, and 532 cm^–1^ is a characteristic band for Fe–C≡N stretching. Two bands are also noticed at 2091 and 2152 cm^–1^, characteristic of the PB’s C≡N stretching modes.^13,20^ The increase of PB content in the freestanding films is also confirmed by the increase in relative intensity between the PB and rGO bands.

### 3.3 Amperometric Detection of H_2_O_2_

**Figure 4:**
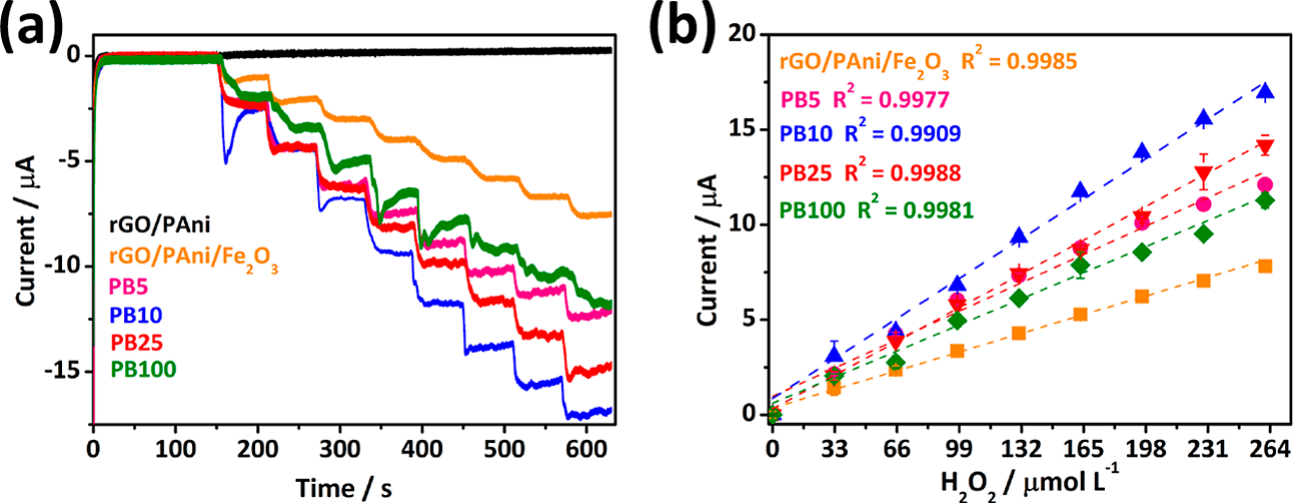
Amperometry signals to assess the sensitivity of freestanding films with increasing H_2_O_2_ additions (a) and the analytical curves (b).

The catalytic performance of PB and PB-analogues for H_2_O_2_ detection is well-known.^14,33,34^ Fe^2+^ species in PB nanoparticles can catalyze the H_2_O_2_ molecules into hydroxyl ions and hydroxyl radicals, similarly to a peroxidase-like activity. We employed these materials as working electrodes in a three-electrode system to explore the feasibility of the free-standing graphene/PB-based composites as current collectors and an electrochemical sensor for H_2_O_2_ detection. Initially, we performed the chronoamperometric detection of H_2_O_2_ (Figure 4a) in PBS buffer (0.1 mol L^–1^) and KCl (0.1 mol L^–1^) at −0.1 V within 33–264 μmol L^–1^ of H_2_O_2_. The tests were carried out with the bare electrodes (rGO/PAni and rGO/PAni/Fe_2_O_3_) and graphene/PB-based electrodes (i.e., PB5, PB10, PB25, and PB100). As expected, the rGO/PAni electrode does not exhibit any detection signal throughout the H_2_O_2_ additions since no H_2_O_2_-sensitive material is present in its composition. The rGO/PAni/Fe_2_O_3_ electrode shows some sensitivity toward H_2_O_2_ due to the presence of iron particles, which possess a high surface area and fast electron transfer properties.^35^ Nevertheless, in comparison, the composite films with PB present contrasting responses with much more pronounced current increments upon additions of H_2_O_2_. The higher chronoamperometric response after eight successive H_2_O_2_ additions is achieved by PB10, with a sharper slope in the analytic curve (Figure 4b) compared to the other samples, resulting in a superior sensitivity.

The most prominent response of the PB10 sample may result from the electrochemical design of the electrode. As observed from SEM, nanometric PB cubes (47 ± 20 nm) homogeneously decorate this electrode, providing many active sites for H_2_O_2_ detection. With the increase of electrodeposition cycles, more PB is formed on the films. However, the particles tend to grow and/or agglomerate. This growth behavior reduces surface area and decreases PB particle contact with the supporting substrate and current collector film. Therefore, even though PB is the H_2_O_2_-sensitive material in the composite film, the sensitivity is not directly related to the amount of PB present on morphological features. Moreover, we have calculated the electrochemically active area (EAA) of the most prominent electrode for H_2_O_2_ detection (PB10) in comparison with the bare electrode (the rGO/PAni/Fe_2_O_3_). The values were achieved throughout CV measurements at different scan rates in K_3_[Fe(CN)_6_]/K_4_[Fe(CN)_6_]. As provided in Figure S7, both electrodes do not show a reversible electrochemical profile, with peak potential separation close to 390 mV for the rGO/PAni/Fe_2_O_3_ electrode and 342 mV for the PB10 electrode at 5 mV s^–1^. Such behavior is inherent to the characteristics of the electrodes. Based on the appropriate Randles–Sevick equation,^36,37^ the EEA of both electrodes was obtained using eq 1
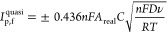
1where *I* is the anodic peak current (A), *n* is the number of electrons involved in the electrochemical reaction, *F* is the Faraday constant (C mol^–1^), *A*_real_ is the EAA, *C* is the K_3_[Fe(CN)_6_]/K_4_[Fe(CN)_6_] concentration, *R* is the universal gas constant, and *T* is the temperature (K). The EEA for the PB10 electrode corresponds to 0.87 cm^2^, 61% higher than the nonmodified electrode (rGO/PAni/Fe_2_O_3_) with a value of 0.54 cm^2^. This result corroborates the importance of modifying the graphene-based electrodes with PB nanoparticles to improve H_2_O_2_ detection.

The LDs and limits of quantification (LQs) were calculated according to IUPAC’s recommendations as three times (LD) and ten times (LQ) the standard deviation (σB) of the blank signal divided by the calibration curve slope (m), obtained with the traditional method of external calibration,^38^ using eqs 2 and 3:
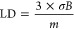
2
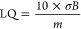
3

Table S1 in Supporting Information presents the analytical values of all freestanding films prepared in this work, and it confirms the best electrochemical detection for sample PB10, with values of 2.00 and 7.00 μM for LD and LQ, respectively. Table 1 compares the best LD value achieved herein (PB10) with some works from literature using graphene/PB-based freestanding electrodes (or paper-like graphene). The LD achieved in this work aligns with other graphene/PB-based electrodes. Further improvements may be performed in our graphene/PB-based electrodes to achieve the best electrochemical results for H_2_O_2_ detection, such as the electrodeposition of PB-analogues or the control of the electrodeposition parameters to increase nucleation and avoid particle growth and agglomeration.

**Table 1:** Comparison for Electrochemical Detection of H_2_O_2_

electrode	LD (μM)	linear range (μM)	references
PBNPS-rGO	5.0	1000–7000	(39)
PB/GCF	2.0	10.0–1310	(40)
Au@PB graphene paper	0.10	1.0–30	(5)
GC/rGO/PB/PTBO	1.5	5.0–600	(41)
PB10	2.0	33.0–264	this work

**Figure 5:**
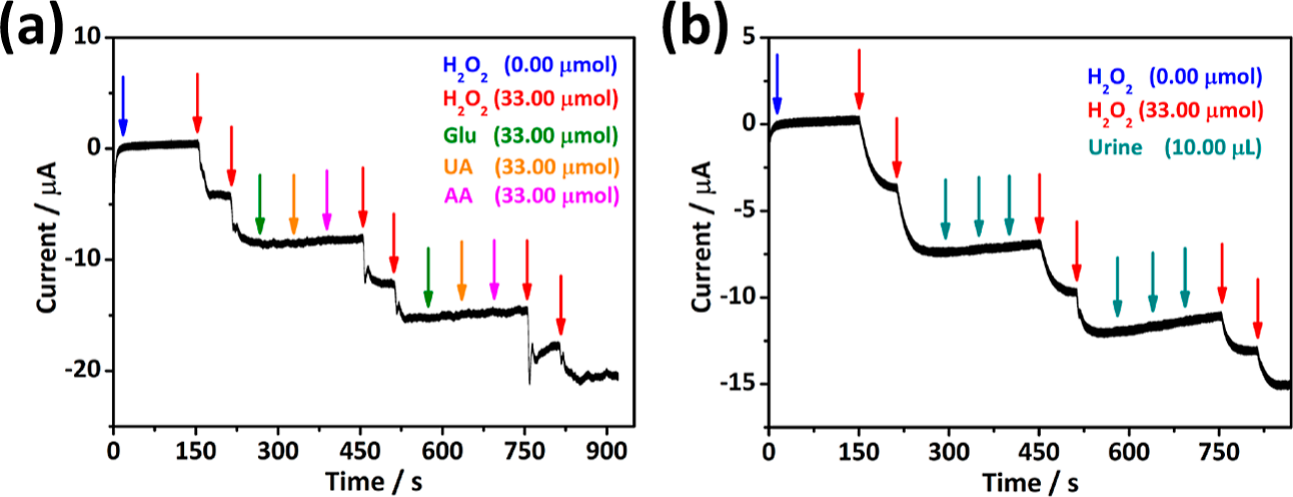
Evaluation of signal disturbance with the intermittent addition of interferents Glu, UA, and AA (a) and artificial urine between additions of H_2_O_2_ using the PB10 freestanding film (b).

We also performed an interference study on the PB10 electrode to assess the selectivity of this freestanding film for chronoamperometric detection of H_2_O_2_. The selectivity is measured by performing H_2_O_2_ chronoamperometric detection in the presence of other compounds, so-called interfering compounds. H_2_O_2_ is a common product of enzymatic reactions. Thus, the H_2_O_2_ detection can be correlated with enzymatic activity. Glucose (Glu), UA, and AA were selected as interfering compounds as they are typically present in the same processes. Figure 5a shows that the intermittent addition of interfering compounds, including Glu, AA, and UA, between the H_2_O_2_ additions has no significant interference effect on the baseline and generated reduction currents. In addition, we controlled the applicability of the PB10 interface for detecting H_2_O_2_ in urine samples containing a broader spectrum of additional interference. Multiple additions of artificial urine (i.e., 10 μL), with high interfering molecule concentrations,^25^ did not cause a disturbance in the detection of H_2_O_2_, as shown in Figure 5b. These evaluations confirm the high selectivity of graphene/PB freestanding films for detecting hydrogen peroxide, even under severe matrix conditions. We have also tested the PB10 electrode endurance throughout multiple additions of H_2_O_2_ every 24 h, as depicted in Figure S8. The electrode exhibited quite similar H_2_O_2_ detection in the first 48 h. Despite the decrease in the electrochemical activity from the third batch of measurements, the electrode exhibited high linearity over consecutive additions of hydrogen peroxide.

The performance for H_2_O_2_ detection of the graphene/PB-based freestanding films synthesized herein may be assigned to fine-tuning the electrochemical growth of PB cubes over the surface of the graphene-based electrode. Nanometric cubes homogeneously distributed over the freestanding film provide more active sites for the catalytic reaction between PB and H_2_O_2_ molecules. More CV cycles for the electrodeposition of PB increase the size of the cubes along with their agglomeration, which compromises the electrochemical detection of H_2_O_2_. The freestanding films prepared herein work both as a current collector and electroactive material for detecting H_2_O_2_, with no need for any additive (binder or conductive material) to improve the electrochemical performance of the electrodes. Finally, progress in the freestanding film process and electrochemical deposition of PB-analogues may be performed to provide more sensitivity for detecting H_2_O_2_ and other analytes.

## 4 Conclusions

Graphene/PB-based freestanding films were prepared by electrochemical deposition of PB cubes over rGO/PAni/Fe_2_O_3_ films. The films synthesized herein work as current collectors and electrochemical sensors for H_2_O_2_. Fe_2_O_3_ nanoparticles in the freestanding films are pivotal to the heterogeneous reaction for PB electrodeposition. Nanometric-sized PB cubes were produced by adjusting the number of CV cycles. The freestanding electrode with cubes around 47 ± 20 nm presented the best chronoamperometric detection of H_2_O_2_, with LD and LQ values of 2.00 and 7.00 μM, respectively, evidencing the structure/properties balance for the best electrochemical performance. The graphene/PB electrodes shown herein pave pathways toward preparing new graphene/PB analogue-based electrodes aiming for application as a chemical sensor for other analytes or energy storage devices.

## Supporting Information Available

Photographic images of rGO/PAni/Fe_2_O_3_ freestanding films; electrochemical profile of PB growth over the graphene-based electrodes for samples PB5, PB10, PB25, and PB100; SEM image of the rGO/PAni film; size distribution of PB cubes in samples PB5, PB10, PB25, PB100, and PB200; cross-sectional images of the freestanding film before and after the electrodeposition of PB particles (PB100); FTIR spectra of the bare electrode (rGO/PAni/Fe_2_O_3_) and samples PB10, PB25, and PB100; anodic peak current vs square root of the scan rate of rGO/PAni/Fe_2_O_3_ and PB10 electrodes—insets: cyclic voltammograms at different scan rates of rGO/PAni/Fe_2_O_3_ and PB10 electrodes; multiple chronoamperometric detections of H_2_O_2_ every 24 h and the corresponding analytical curve; and electrochemical performance for H_2_O_2_ detection of the freestanding films synthesized in this work (PDF)
