# Supplementary material for: Freestanding Films of Reduced Graphene Oxide Fully Decorated with Prussian Blue Nanoparticles for Hydrogen Peroxide Sensing
Source: ACS Omega. 2024 Jul 10;9(29):31569–77. doi: 10.1021/acsomega.4c01457 (PMC11270561; doi:10.1021/acsomega.4c01457)
Supplement: Supplementary file 1 — ao4c01457_si_001.pdf [file ao4c01457_si_001.pdf]

Supporting information

Freestanding Films of Reduced Graphene Oxide Fully Decorated with Prussian Blue Nanoparticles for Hydrogen Peroxide Sensing

*Vitor H.N. Martins†; Monize M. da Silva†; Daniel A. Gonçalves†; Volker Presser‡¥§, Samantha Husmann‡; Victor H.R. Souza†**

† Faculty of Exact Science and Technology, Universidade Federal da Grande Dourados, Dourados, MS, Brazil

‡ INM – Leibniz Institute for New Materials, Campus D2-2, 66123, Saarbrücken, Germany

¥ Department of Materials Science & Engineering, Saarland University, Campus D2 2, 66123, Saarbrücken, Germany

§ Saarene – Saarland Center for Energy Materials and Sustainability, Campus C4 2, 66123 Saarbrücken, Germany

* Corresponding author: victorsouza@ufgd.edu.br;

Supporting information

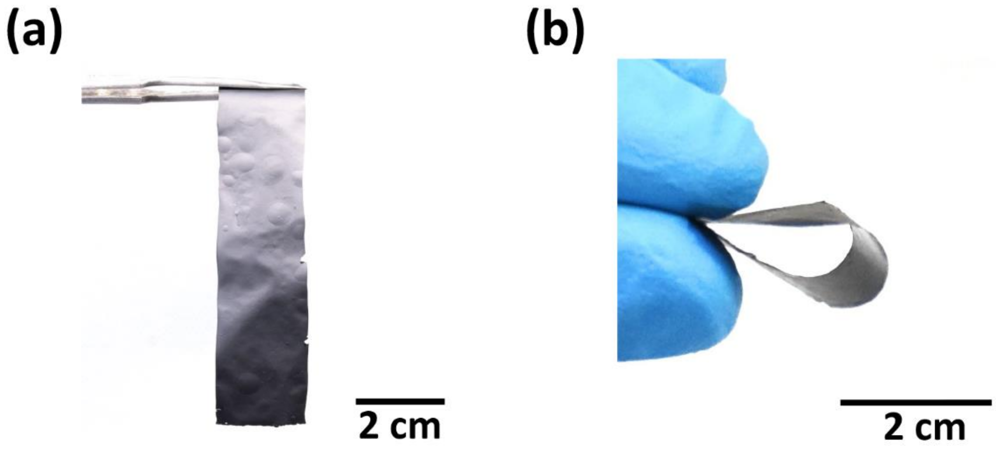

Figure S1. Photographic images of rGO/PAni/Fe_2O_3 freestanding films (a) as-synthesized, and (b) under mechanical deformation.

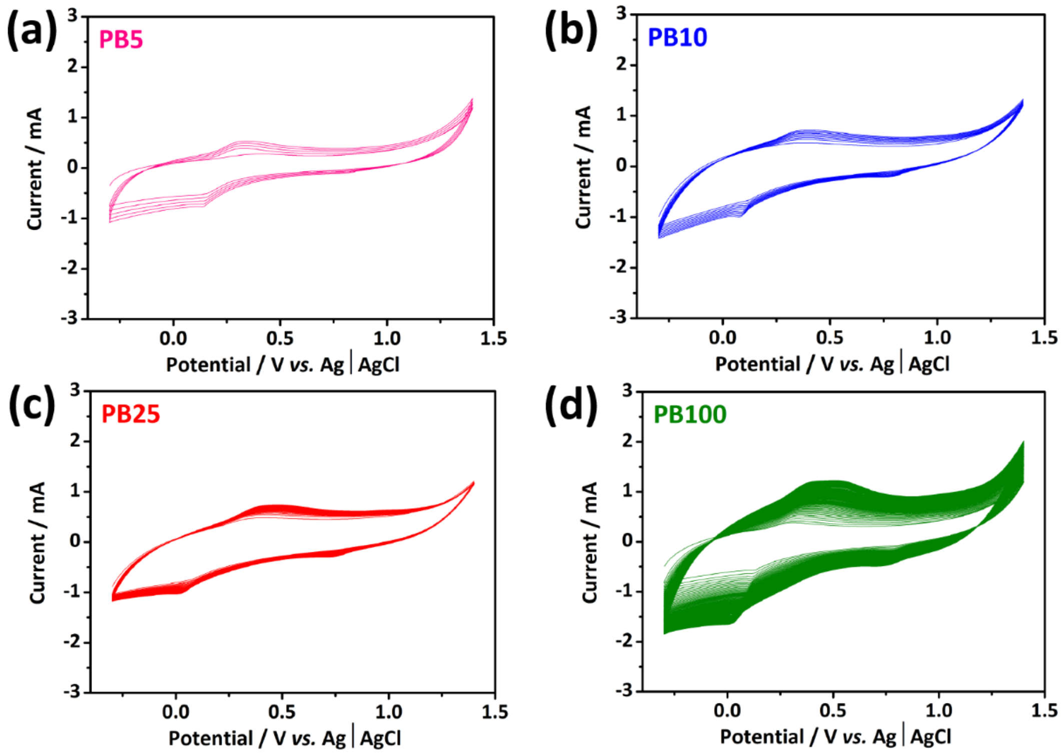

Figure S2. Electrochemical profile of PB growth over the graphene-based electrodes for samples PB5(a), PB10 (b), PB25 (c), and PB100 (d).

Supporting information

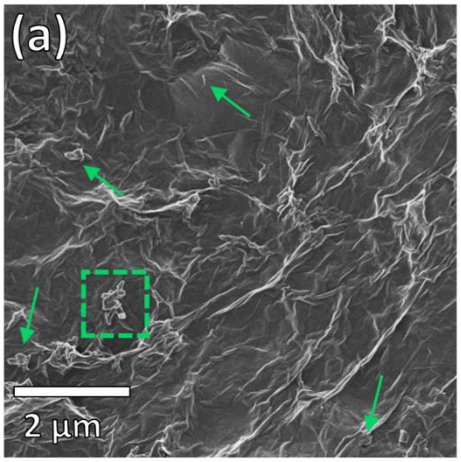

Figure S3. SEM image for rGO/PAni film.

Supporting information

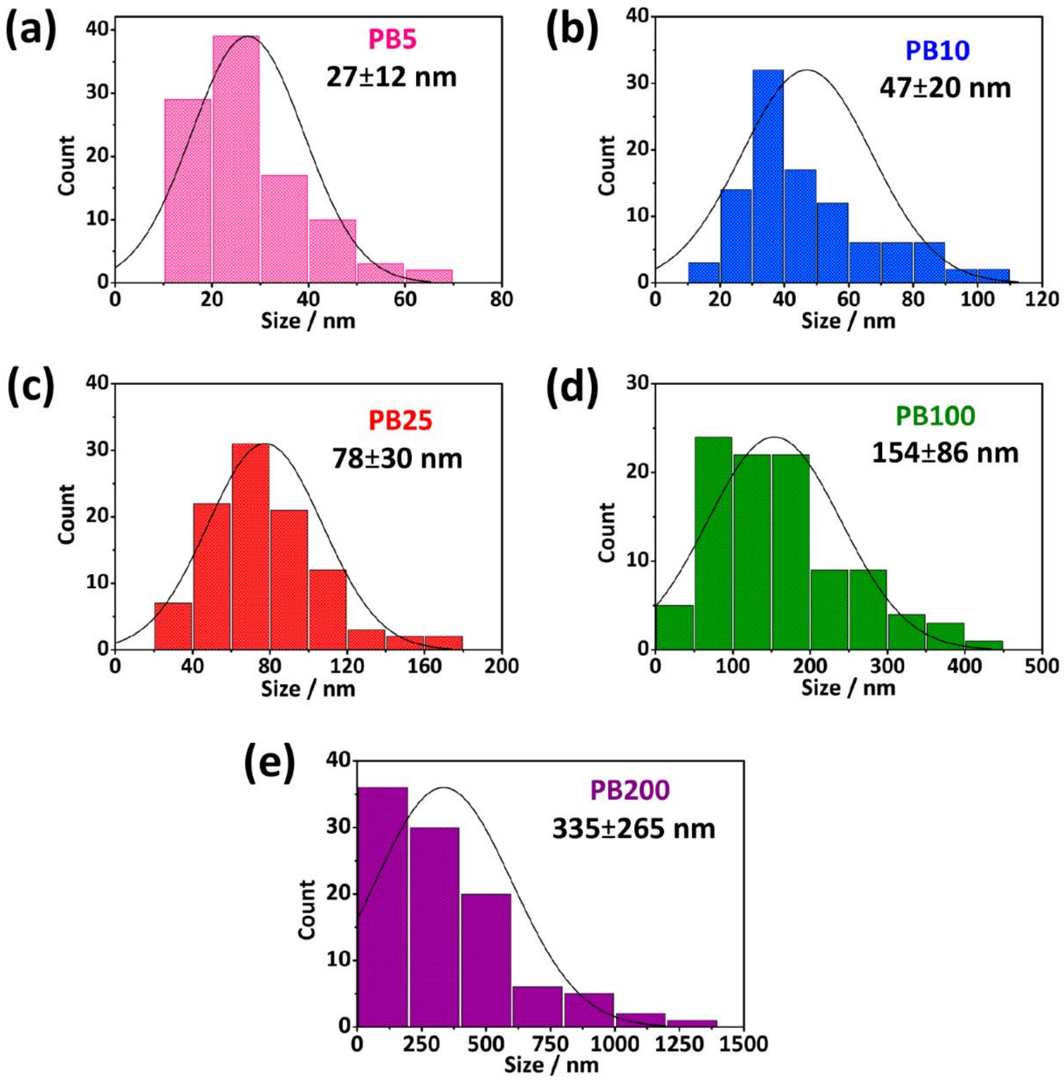

Figure S4. Size distribution of PB cubes in samples PB5 (a), PB10 (b), PB25 (c), PB100 (d), and PB200 (e).

Supporting information

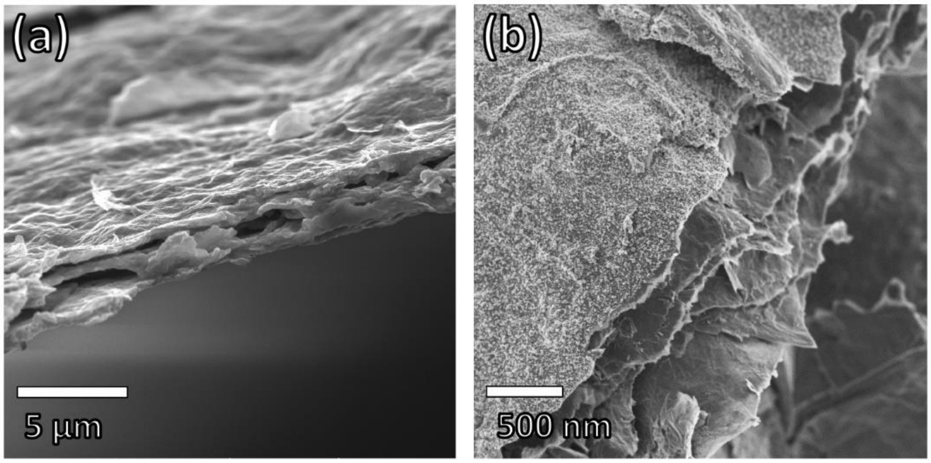

Figure S5. Cross-sectional images of the freestanding film before (a) and after (b) the electrodeposition of PB particles (PB100).

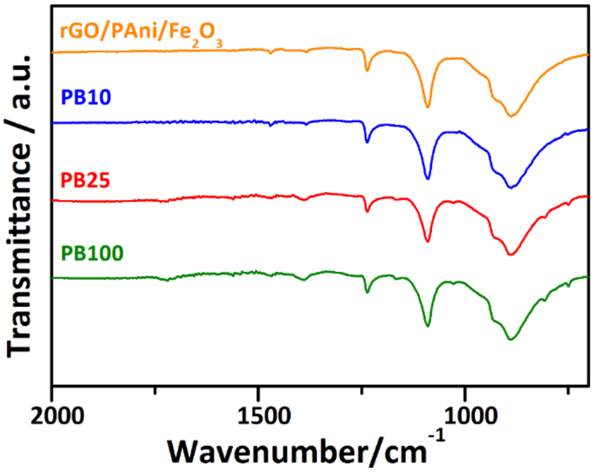

Figure S6. FTIR spectra of the bare electrode (rGO/PAni/Fe_2O_3) and samples PB10, PB25, and PB100.

The FTIR spectra of the bare sample (rGO/PAni/Fe_2O_3) and PB10, PB25, and PB100 electrodes are depicted in Figure S5. The three main bands observed in all samples at 1236, 1090, and 888 cm^{-1} correspond to $\nu_{C–N}$ in benzenoid rings of PAni; $\nu_{–NH}{}^{+\bullet}$ of PAni, indicating the presence of the polymer in emeraldine salt protonated form, and $\gamma_{C–H}$ of both 1,4 and 1,2,4 rings. All bands confirm the presence of PAni in its conductive form.[1-5]

Supporting information

1. Trchová, M.; Stejskal, J. Polyaniline: The Infrared Spectroscopy of Conducting Polymer Nanotubes (IUPAC Technical Report). Pure Appl. Chem. 2011, 83, 1803−1817.
2. Quillard, S.; Louarn, G.; Lefrant, S.; Macdiarmid, A. G. Vibrational Analysis of Polyaniline: A Comparative Study of Leucoemeraldine, Emeraldine, and Pernigraniline Bases. Phys. Rev. B: Condens. Matter Mater. Phys. 1994, 50, 12496−12508.
3. Ping, Z.; Nauer, B. G. E.; Neugebauer, H.; Theiner, J.; Neckel, A.; Neckel, A. Protonation and Electrochemical Redox Doping Processes of Polyaniline in Aqueous Solutions: Investigations Using In Situ FTIR-ATR Spectroscopy and a New Doping System. J. Chem. Soc., Faraday Trans. 1997, 93, 121−129
4. Kang, E.; Neoh, K. G.; Tan, K. L. Polyaniline: A Polymer with Many Interesting Intrinsic Redox States. Prog. Polym. Sci. 1998, 23, 277−324.
5. Martins, V. H. N., Siqueira, N. M. S., Fonsaca, J. E. S., Domingues, S. H. & Souza, V. H. R. Ternary Nanocomposites of Reduced Graphene Oxide, Polyaniline, and Iron Oxide Applied for Energy Storage. ACS Applied Nano Materials 2021, 4, 5553-5563.

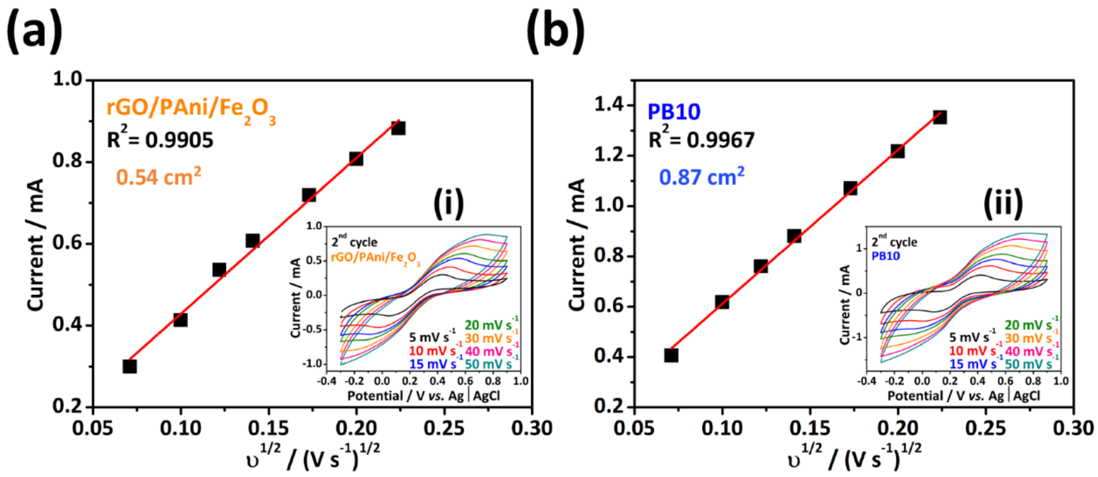

Figure S7 – Anodic peak current *vs.* square root of the scan rate of rGO/PAni/Fe_2O_3 (a) and PB10 (b) electrodes. Insets: Cyclic voltammograms at different scan rates of rGO/PAni/Fe_2O_3 (i) and PB10 (ii) electrodes.

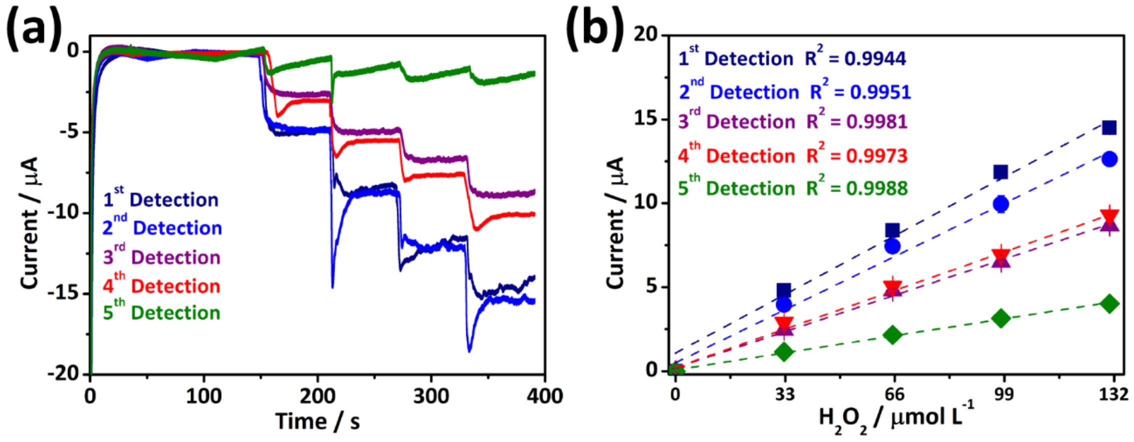

Figure S8. (a) Multiple chronoamperometric detections of H_2O_2 every 24 hours, and (b) the analytical curve calculated from (a).

Table S1. Electrochemical performance for H_2O_2 detection of the freestanding films synthesized in this work.

Sample	Limit of detection (μM)	Limits of quantification (μM)
**rGO/PAni/Fe_2O_3**	25.0	85.0
**PB5**	5.0	15.0
**PB10**	2.0	7.0
**PB25**	4.0	10.0
**PB100**	20.0	80.0
